# Evaluation of Mitral Apparatus Blood Cyst: A Case Report and Review of Literature

**DOI:** 10.7759/cureus.5812

**Published:** 2019-10-01

**Authors:** Dipesh Ludhwani, Belaal Sheikh, Yahya Sheikh

**Affiliations:** 1 Internal Medicine, Rosalind Franklin University of Medicine and Science, North Chicago, USA; 2 Internal Medicine, Baruch College, New York, USA

**Keywords:** mitral valve, cyst, stress testing

## Abstract

Intracardiac blood cysts (ICBC) are cardiac pseudoneoplasm commonly seen in infants below two months of age. ICBC typically resolve spontaneously; however, they can sometimes persist in adults and can cause detrimental consequences. A 47-year-old female presented to our facility with complaints of chest pain and was found to have an incidental subvalvular chordal mitral apparatus echolucent mass on transthoracic echocardiogram (TTE). A stress echocardiography was performed, which revealed transient left ventricle outflow tract (LVOT) obstruction in the absence of anginal symptoms. A cardiac magnetic resonance imaging (MRI) showed no evidence of increased mass enhancement confirming the diagnosis of a benign blood cyst of mitral apparatus. The cyst was treated conservatively with carvedilol to prevent worsening of exertional LVOT obstruction. A follow-up study done at six months showed stable exertional hemodynamics. There is no general consensus while managing ICBC. In asymptomatic, non-surgical patients stress echocardiography can offer valuable information by assessing the hemodynamic implications resulting from the cyst.

## Introduction

Primary cardiac tumours (PCT) are rare, with an estimated incidence of less than 0.1% [1]. Most PCT are predominantly benign, with myxoma being the most frequent type. Cardiac pseudoneoplasms are non-neoplastic inflammatory excrescences mimicking PCT radiologically, or clinically. The five well known pseudoneoplastic tumours of the heart include (i) inflammatory myofibroblastic tumour, (ii) calcified amorphous tumour, (iii) hamartoma of mature cardiac myocytes, (iv) mesothelial/monocytic incidental cardiac excrescences, and (v) lipomatous hypertrophy of the atrial septum [2]. Intracardiac blood cyst (ICBC), a non-organized collection of blood lined by endothelial cells is typically classified as a cardiac pseudoneoplasm. ICBCs are usually single, fibrotic structures rooted on the endocardium of atrioventricular valves (mitral valve is the most common site). Congenital ICBCs is often seen in 50% of infants at the time of necropsy below two months of age [3].Though relatively common in newborns, blood cysts typically disappear spontaneously and are a rare entity in adults. We present one such case of a 47-year-old healthy woman who presented with chest pain and was found to have an incidental mitral valve blood cyst causing transient left ventricle outflow tract (LVOT) obstruction, which we worked up with exercise stress echocardiography.

## Case presentation

A 47-year-old Caucasian female with a known medical history of essential hypertension being treated with labetalol came to the emergency department (ED) with complaints of non-exertional, intermittent (lasting one to two minutes), 3/10, left-sided chest pain which started three days ago. At the time of presentation, the patient was asymptomatic. She was afebrile with a blood pressure of 188/90 mmHg, heart rate of 73 beats per minute, respiratory rate of 16 per minute and normal oxygen saturation levels. Physical examination revealed a faint 1/6 systolic murmur which accentuated to 2/6 in intensity when she was made to go from crouching to standing. The remainder of the physical exam was unremarkable. The patient denied any pertinent family history. She was a former smoker and reported no allergies. Laboratory studies disclosed normal serum chemistry with normal troponin and brain natriuretic peptide (BNP) levels. Electrocardiogram (EKG) showed normal sinus rhythm with no ST-T wave changes. A chest radiograph identified borderline cardiomegaly with no evidence of infection or pulmonary vascular congestion. 

**Figure 1:**
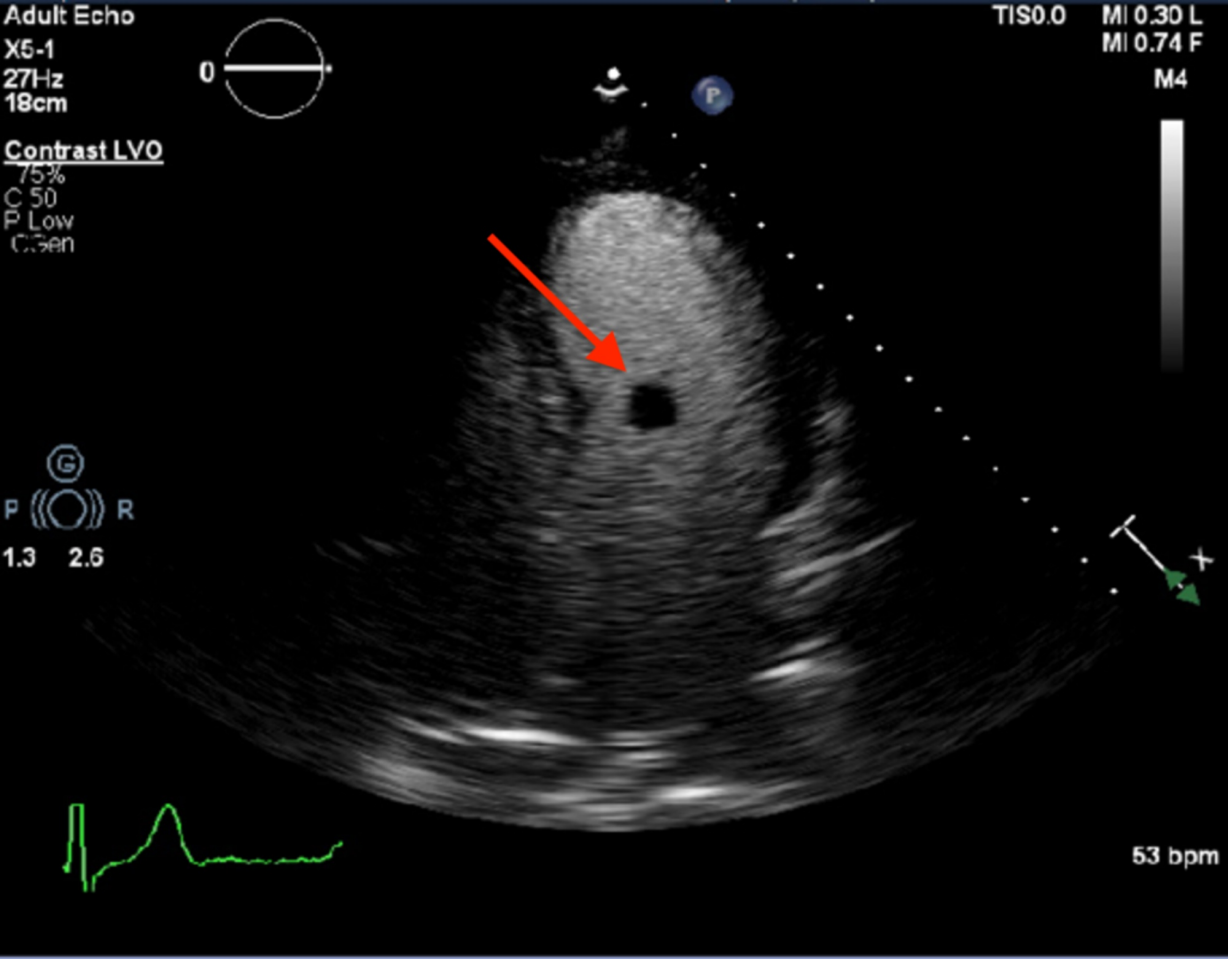
Apical four chamber view of two-dimensional transthoracic echocardiogram at the time of presentation An Echocardiogram showing 11 x 9.5 mm circular echolucent spherical mobile mass at the subvalvular mitral valve apparatus, on the left ventricular aspect (red arrow).

**Figure 2:**
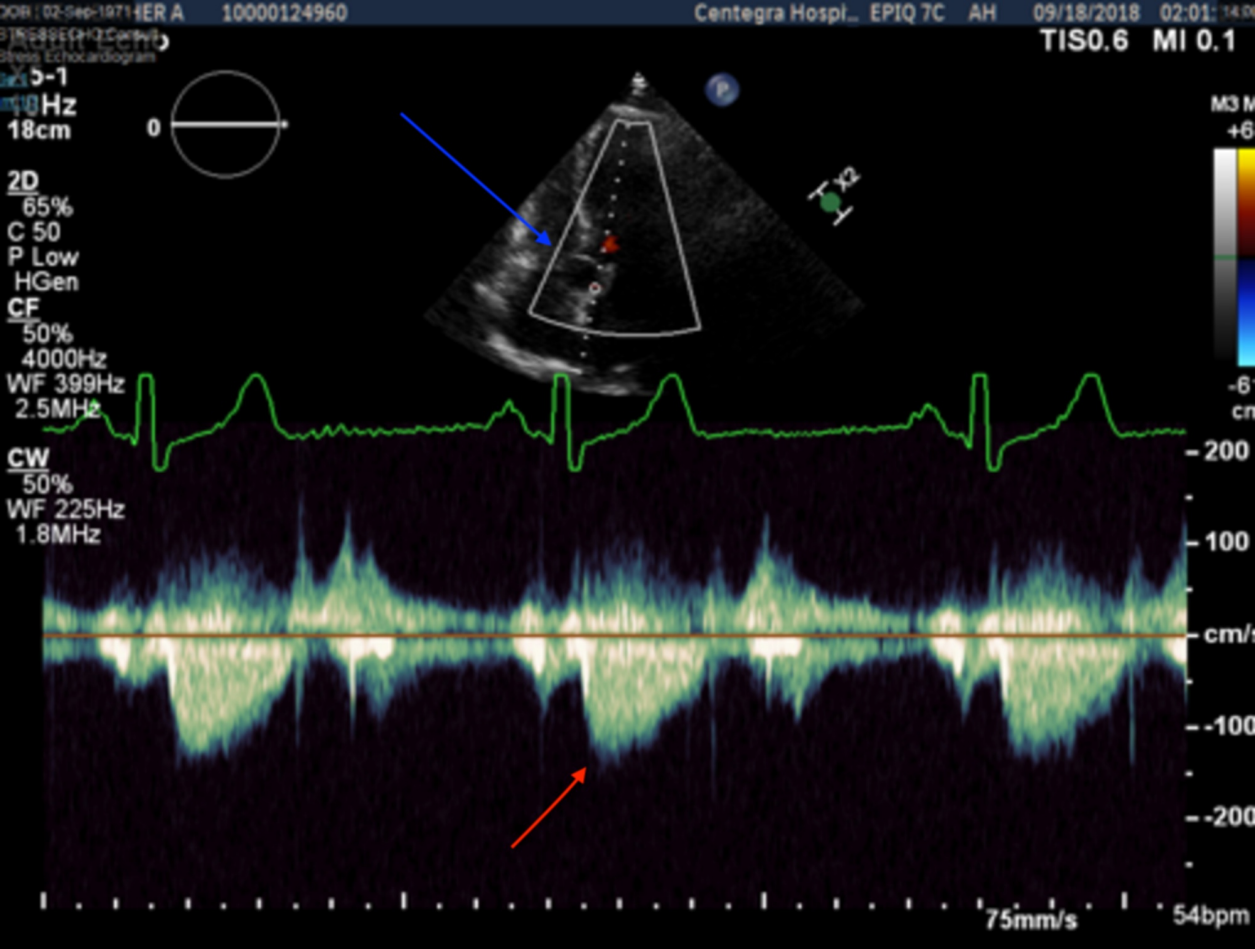
Stress echocardiography aortic view showing mitral cyst (blue arrow) and normal peak velocity with no left ventricle outflow tract obstruction pre-exercise (red arrow)

**Figure 3:**
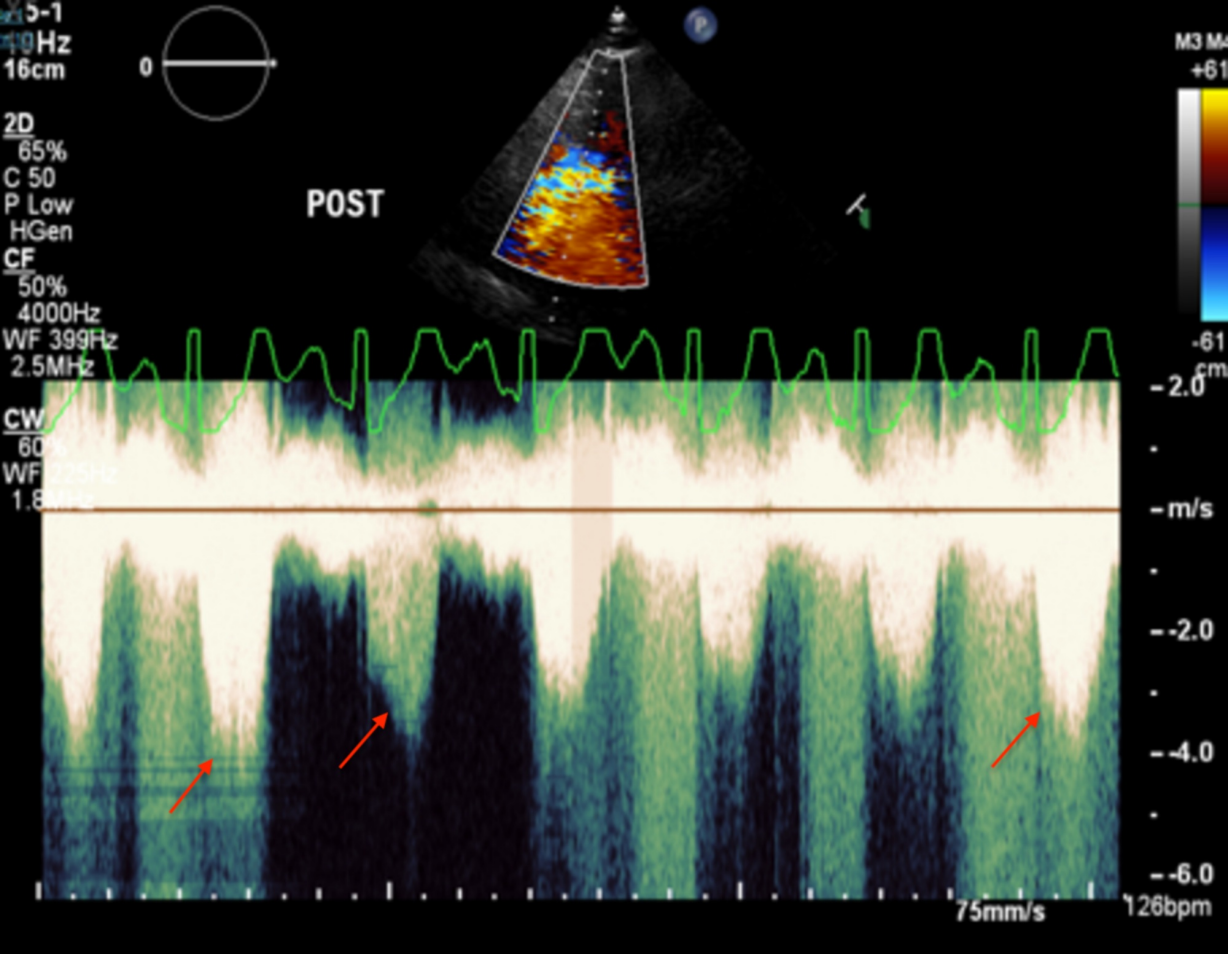
Post-exercise echocardiography showing late peaking dagger shaped transient left ventricle outflow tract obstruction (red arrows)

A transthoracic echocardiogram (TTE) was pursued, which revealed normal left ventricular (LV) size and function. LVOT appeared normal without sub-valvular ridge or asymmetric septal hypertrophy. Mitral valve demonstrated normal-appearing leaflets; however, there was a large centrally echolucent sub-valvular chordal cyst greater than 1 cm in the largest diameter (Figure 1). Due to concerns of possible exertional LVOT obstruction, an exercise stress echocardiography was scheduled. Labetalol was switched to carvedilol due to the latter’s negative inotropy and better heart rate control. During exercise stress echocardiography, the patient exercised to stage five Bruce protocol achieving 14.5 metabolic equivalents (METs). There was no suggestion of obstruction on doppler at rest however, transient LVOT obstruction was noted with exercise. The patient had an appropriate heart rate and blood pressure response to stress with no signs of coronary ischemia or regional wall motion abnormalities. (Figures 2-3). 

A cardiac magnetic resonance imaging (MRI) showed no evidence of increased enhancement confirming the diagnosis of a benign subvalvular blood cyst of the mitral apparatus. In light of asymptomatic nature of the condition, mild to moderate LVOT obstruction at peak exertion in a beta-blocker deprived state, a conservative strategy was opted after multidisciplinary discussion with cardiothoracic surgery. The patient was resumed on carvedilol and was advised to maintain adequate hydration to avoid worsening of LVOT obstruction due to dehydration. A surveillance stress echocardiogram done during six months follow-up visit showed stable exertional hemodynamics.

## Discussion

ICBCs are diverticula resulting from invagination of atrial endothelium into atrioventricular valves. Histologically the cyst walls are made up of fibrous connective tissue either formed by mucous degeneration or calcification. Congenital ICBC often results from lacunar remanence created during the embryological development of atrioventricular valves. Disturbed blood flow can lead to enlargement of blood cysts which against the norm of spontaneous disappearance can persist into adulthood. Other theories such as hematoma formation, vessel ectasia or dilation, and abnormal development of cardiac mesothelial progenitor cells have also been postulated [4]. Acquired blood cysts have also been reported after cardiac catheterization, valvular surgeries, and blunt force trauma. 

The clinical course of ICBC can vary depending on the location and size of the cyst. Majority of the adults are asymptomatic and are diagnosed incidentally. LVOT obstruction from ICBC can result in exertional dyspnea or syncope [5]. Isolated cases of thromboembolic stroke, retinal or coronary artery embolism due to ruptured ICBC have also been reported in the literature [6]. TTE remains the first-line diagnostic test for ICBC [7]. The cystic hypoechoic appearance of a valvular mass during TTE in absence of other obvious clinical illnesses should raise suspicion for ICBC. Enhanced cardiac MRI with contrast material can help assess tissue properties such as vascularity and fibrosis [8]. The malignant cardiac mass tends to be large-sized (greater than 5 cm), irregular, invading surrounding structures, contrast-enhancing and exhibiting tissue heterogenicity on T1 and T2 weighted images [9]. Once detected by echocardiography, conjugate use of cardiac MRI can help determine the benign nature of the cardiac mass.

In our case, given the episodic nature of patient’s symptoms a decision was made to perform an exercise stress echocardiogram to establish hemodynamic consequences from the mass. Though there have been previously reported cases of LVOT obstruction, to the best of our knowledge this is the first case in which ICBC was worked up with exercise stress echocardiography. Stress echocardiography is a non-invasive valuable tool which can help evoke underlying dynamic LVOT and ascertain the need for surgical removal. Due to the patient’s reluctance to have surgical intervention and asymptomatic nature of her condition at maximal stress, the patient was treated conservatively with optimum medical management and surveillance echocardiogram. 

Management of ICBC is controversial. Due to the lack of general consensus or guidelines, management varies based on the nature of the presenting symptoms and surgical appropriateness. Earlier recommendation by Paşaoğlu et al. was to resect all valvular cystic tumor to achieve a definitive diagnosis [10],however, recent reports have suggested conservative management for asymptomatic cases [11]. We recommend surgical excision for all patients with active symptoms; evidence of a hemodynamic compromise or valvular insufficiency on cardiac imaging. Thromboembolic cases resulting in systemic manifestation warrant surgical resection. Anticoagulation can be considered in patients who are poor surgical candidates however, the role remains debatable [12]. In asymptomatic patients with no evidence of hemodynamic compromise or LVOT obstruction conservative management is reasonable. Moving forward incorporating stress echocardiography in routine practice, when tolerated by the patient can provide further insight into the patient’s symptomology and help direct appropriate therapy against benign blood cyst of the heart.

## Conclusions

ICBCs are diverticula resulting from invagination of atrial endothelium into atrioventricular valves. The clinical course of ICBC varies depending on the degree of hemodynamic compromise. In symptomatic cases with dyspnea, stroke, and coronary artery embolism, surgical removal of the cyst in warranted. Exercise stress echocardiography offers an alternate approach to assess hemodynamic consequences from ICBC.

## References

[1] Lam KY, Dickens P, Chan AC (1993). Tumors of the heart. A 20-year experience with a review of 12,485 consecutive autopsies. Arch Pathol Lab Med.

[2] Miller DV, Tazelaar HD (2010). Cardiovascular pseudoneoplasms. Arch Pathol Lab Med.

[3] Zimmerman KG, Paplanus SH, Dong S, Nagle RB (1983). Congenital blood cysts of the heart valves. Hum Pathol.

[4] Park MH, Jung SY, Youn HJ, Jin JY, Lee JH, Jung HO (2012). Blood cyst of subvalvular apparatus of the mitral valve in an adult. J Cardiovasc Ultrasound.

[5] Arnold IR, Hubner PJ, Firmin RK (1990). Blood filled cyst of the papillary muscle of the mitral valve producing severe left ventricular outflow tract obstruction. Br Heart J.

[6] Khan T, El-Sharkawy S, Schafer F (2012). A heart within the heart--a rare congenital cause of an embolic stroke. BMJ Case Rep.

[7] Xie SW, Lu OL, Picard MH (1992). Blood cyst of the mitral valve: detection by transthoracic and transesophageal echocardiography. J Am Coll Cardiol.

[8] Hundley WG, Bluemke DA, Finn JP (2010). ACCF/ACR/AHA/NASCI/SCMR 2010 expert consensus document on cardiovascular magnetic resonance: a report of the American College of Cardiology Foundation Task Force on expert consensus documents. J Am Coll Cardiol.

[9] Sparrow PJ, Kurian JB, Jones TR, Sivananthan MU (2005). MR Imaging of cardiac tumors. RadioGraphics.

[10] Paşaoğlu I, Doğan R, Nazli N, Güngen Y, Bozer AY (1991). Blood cyst originating from tricuspid septal leaflet. J Cardiovasc Surg (Torino).

[11] Pelikan HM, Tsang TS, Seward JB (1999). Giant blood cyst of the mitral valve. J Am Soc Echocardiogr.

[12] Sun F, Ren W, Bi W, Zhang Y, Guo B. (2013). A “balloon” on the mitral valve. J Am Coll Cardiol.

